# Editorial: Glial Cells: Managers of Neuro-Immunity

**DOI:** 10.3389/fncel.2016.00060

**Published:** 2016-03-23

**Authors:** Carlos Barcia, Gilles J. Guillemin, James F. Curtin, Jeffrey M. Zirger

**Affiliations:** ^1^Department of Biochemistry and Molecular Biology, Institut de Neurociències, Universitat Autònoma de BarcelonaCerdanyola del Vallès, Spain; ^2^Department of Biomedical Sciences, Faculty of Medicine and Health Sciences, Macquarie UniversitySydney, NSW, Australia; ^3^School of Food Science & Environmental Health, Dublin Institute of TechnologyDublin, Ireland; ^4^U.S. Centers for Disease Control and PreventionAtlanta, GA, USA

**Keywords:** glia, neuroinflammation, neuroimmunology, microglia, astroglia, T cells

After many decades of study in the field of Neuroscience that were mostly centered on the neuron there is a mounting interest in the study of the function of the glial cells in many aspects and functions of the central nervous system. The involvement of glial cells in neuroimmunity is one of the critical pieces within this puzzle, and one that entails great complexity. An increasing number of publications shows that resident astroglia and microglia are the real managers of immune responses, orchestrating chemokine and cytokine release, blood cell infiltration, and promotion of angiogenesis, etc. Moreover, each disease and neuroinflammatory scenario seems to have its own distinct biochemical characteristics and glial phenotype. Classical definitions of resting and activated microglial cells or pro-inflammatory and anti-inflammatory phenotypes are recognized today as oversimplified models of glial cell functions and have since been surpassed by more defined and precise characterizations. The present Frontiers Research Topic (FRT) is a great example of this, since the study of different scenarios reflects diverse modes of glial activation and distinct complexities.

We present here a selection of articles, both original research and reviews, solving clinical, and basic aspects of the biology of glial cells in neuro-inflammatory and neuro-immune scenarios.

A good number of manuscripts in this FRT shows the importance of glial cell-derived inflammation on neurodegenerative diseases. Particularly, Ben Haim and colleagues, from Escartin's lab, show a compelling review on the peculiar, and still poorly understood, roles of astrocytes in neurodegenerative diseases, unfolding the signaling pathways toward reactivity (Ben Haim et al.). Von Bernhardi et al., review the roles of glial cells in neurodegeneration, but focused on Alzheimer's disease and particularly discussing the effects of the cytokine TGFβ (von Bernhardi et al.). Herrera et al. center their attention on another major neurodegenerative disorder, Parkinson's disease, and how stress and glucocorticoids may interact and play important roles in modulating microglial activation (Herrera et al.). Yuste and colleagues, give us an interesting view of the role of nitric oxide in neurodegenerative diseases from a glial-derived inflammatory perspective (Yuste et al.). Vieira et al., review the glial reaction triggered in multiple system atrophy (MSA) focused on the α-synuclein-mediated activation (Vieira et al.).

Other papers are centered on Multiple Sclerosis. Crowley et al., for instance, present an original paper characterizing the roles of Baclofen, a well-known GABA B receptor agonist used clinically, for regulating TLR3 and TLR4 signaling in murine glial cells and peripheral monocytes obtained from Multiple Sclerosis patients (Crowley et al.). Huseby and colleagues review another aspect, focusing their manuscript on the amplification of the neuroinflammatory response due to glial cells-T cell interactions (Huseby et al.). Almolda et al. from González and Castellano's lab, also review the topic of glia-lymphocyte crosstalk but compellingly covering other pathological scenarios, suggesting that microglial cells are able to acquire a phenotype of dendritic cells (Almolda et al.).

We also include articles reflecting that inflammatory glial response is involved in mental and psychiatric alterations, which include frontotemporal dementia associated with amyotrophic lateral sclerosis (ALS), reviewed by Radford et al. and the pathogenesis of delirium, reviewed by Sfera et al.

Due to its patent roles in neuro-immunity, microglial cells and brain macrophages are the main protagonists of many of the papers included in this FRT. We would like to highlight the appealing work on the *in vivo* characterization of microglial engulfment of dying neurons presented by Morsch et al., which represents a fine piece of basic science (Morsch et al.). From a clinical point of view, Spanos et al., review the roles of microglia in brain infection, particularly in CNS tuberculosis and how this may affect future therapeutic strategies (Spanos et al.). Following the focus on microglia, Perrotta et al., present an interesting perspective article on the importance of the microglia-glioma cells crosstalk in hormone and immune-derived response in glioma (Perrotta et al.). Particularly important for glioma is the modification of the phenotype, which varies from the classically activated to pro-tumoral phenotype. In this context of phenotype modulation, Kopitar-Jerala reviews the novel role of the cystatin, statin B, in modulating microglial cells toward a pro and anti-inflammatory response (Kopitar-Jerala). In a different scenario, López-Pedrajas et al. report here that cocaine-treated rats show microglial activation in the cerebellum, suggesting that glial reaction may have important implications in motor control during drug addiction (Lopez-Pedrajas et al.).

Importantly, for the field of the long-term maintenance of the neuro-immune response, an interesting original article, Cao et al., reports that prior activation of microglia during embryo development may have consequences in the susceptibility to inflammation in the life of the newborns (Cao et al.). This advocates for the importance of glial cells driving lasting immune responses. Finally, Bas et al., report that infiltrated macrophages participate in the repair of spiral ganglion cells and neurons forming the cochlear nerve, having important implications for successful cochlear implant surgery (Bas et al.).

We believe that this comprehensive collection of articles contains valuable information that will contribute to the knowledge on how glial cells drive the management of neuro-immunity. As the editorial team, we hope you enjoy reading, *Carlos Barcia, Gilles J Guillemin, James F. Curtin, Jeffrey M. Zirger*.

## Author contributions

All authors listed, have made substantial, direct and intellectual contribution to the work, and approved it for publication.

### Conflict of interest statement

The authors declare that the research was conducted in the absence of any commercial or financial relationships that could be construed as a potential conflict of interest.

